# Understanding the Correlation between Metabolic Regulator SIRT1 and Exosomes with CA-125 in Ovarian Cancer: A Clinicopathological Study

**DOI:** 10.1155/2022/5346091

**Published:** 2022-04-20

**Authors:** Sraddhya Roy, Ananya Das, Manisha Vernekar, Syamsundar Mandal, Nabanita Chatterjee

**Affiliations:** ^1^Department of Receptor Biology and Tumor Metastasis, Chittaranjan National Cancer Institute, Kolkata, India; ^2^Department of Gynaecological Oncology, Chittaranjan Cancer Hospital, Kolkata, India; ^3^Department of Epidemiology and Biostatistics, Chittaranjan National Cancer Institute, Kolkata, India

## Abstract

**Background:**

Ovarian cancer (OvCa), the deadliest gynaecological malignancy, is associated with poor prognosis and high mortality rate. Ovarian cancer has been related with CA-125 and metabolic reprogramming by SIRT1 leading to metastasis with the involvement of exosomes.

**Methods:**

Clinicopathological data of OvCa patients were collected to perform the analysis. Patients' samples were collected during surgery for immunohistochemistry and flow cytometric analysis of SIRT1, HIF-1*α*, exosomal markers (CD81 and CD63), ki-67, and PAS staining for glycogen deposition. Adjacent normal and tumor tissues were collected as per the CA-125 levels.

**Results:**

CA-125, a vital diagnostic marker, has shown significant correlation with body mass index (BMI) (*P* = 0.0153), tumor type (*P* = 0.0029), ascites level, ascites malignancy, degree of dissemination, tumor differentiation, FIGO stage, TNM stage, laterality, and tumor size at *P* < 0.0001. Since significant correlation was associated with BMI and degree of dissemination, as disclosed by IHC analysis, metabolic marker SIRT1 (*P* = 0.0003), HIF-1*α* (*P* < 0.0001), exosomal marker CD81 (*P* < 0.0001), ki-67 status (*P* = 0.0034), and glycogen deposition (*P* <0.0001) were expressed more in tumor tissues as compared to the normal ones. ROC analysis of CA-125 had shown 327.7 U/ml has the best cutoff point with 82.4% sensitivity and specificity of 52.3%. In addition, Kaplan-Meier plots of CA-125 (*P* < 0.0001), BMI (*P* = 0.001), degree of dissemination (*P* < 0.0001), and ascites level (*P* <0.0001) reflected significant correlation with overall survival (OS). Upon multivariate Cox-regression analysis for overall survival (OS), BMI (*P* = 0.008, HR 1.759, 95% CI 1.156-2.677), ascites malignancy (*P* = 0.032, HR 0.336, 95% CI 0.124-0.911), and degree of dissemination (*P* = 0.004, HR 1.994, 95% CI 1.251-3.178) were significant proving to be independent indicators of the disease.

**Conclusion:**

Clinicopathological parameters like BMI, degree of dissemination, and ascites level along with CA-125 can be prognostic factors for the disease. Levels of CA-125 can depict the metabolic and metastatic factors. Thus, by targeting SIRT1 and assessing exosomal concentrations to overcome metastasis and glycogen deposition, individualized treatment strategy could be designed. In-depth studies are still required.

## 1. Introduction

Ovarian cancer (OvCa) is the third most common gynaecological malignancy after cervical and uterine cancer with the highest mortality and morbidity rate among all gynaecological malignancies [1, 2]. Limitation of early diagnosis is the major reason for poor survival and increased mortality rates among the patients [3]. Late diagnosis is the result of nonspecific and inappropriate symptoms which are mostly misinterpreted as normal gastrointestinal and urinary symptoms. In addition to this, several other factors possess the risk of developing OvCa like age, menopausal status, body mass index (BMI), parity, and family history [4]. As per World Health Organization (WHO) classification, 60% of all primary OvCa is of epithelial origin, followed by germ cell (30%) and sex cord stromal tumor (8%). Carcinomas of epithelial origin is the most frequent form of OvCa having 80-85% occurrence rate and is subcategorized depending on the proliferation rate of epithelial cells and on histopathological types [5]. Epithelial OvCa (EOC) is a heterogeneous group of neoplasms which has been categorized under various histological subtypes, namely, serous, mucinous, endometrioid, clear cell, transitional cell tumors (Brenner tumors), carcinosarcoma, mixed epithelial tumor, and undifferentiated carcinoma [6]. India ranks the highest in mortality rate among the Asian countries whereas incidences among young females have reduced in Europe and North America [7]. The age-adjusted OvCa occurrence rates in India lie between 0.9 to 8.4 per 100,000 population distributed in different parts of the country [8]. It has been estimated that by the end of 2020, there would be 59,276 new OvCa cases in India, and it has been assessed that the rate of incidences would increase by 55% by the end of 2035 while mortality rate would be uplifted by 67% [9]. In Indian scenario, where the patients are diagnosed at a later stage and with bleak chances of good prognosis, there is an absolute necessity to assess the disease at an early stage to improve the survival.

Accurate monitoring of the disease is hindered by local invasion of the tissues deep within the pelvis and peritoneal seeding leads to metastasis, thereby making early diagnosis difficult to assess. Thus, there is a need to detect the disease with preferably good diagnostic and prognostic factor. A wide range of clinical and histopathological characteristics have been exhibited by the primary OvCa lesions. Out of the various clinicopathological parameters, serum cancer/carbohydrate antigen-125 (CA-125), a glycoprotein synthesized by neoplastic OvCa cells, has been proven to be a potent tumor marker, which is closely associated with tumor burden and easy to assay [10].

Persistent efforts are being conducted to assess the tumor molecular markers like ki-67 (a proliferation marker), p53, estrogen receptors, progesterone receptors, and E-cadherin (E-cad) of EOC by immunohistochemical (IHC) analysis [11, 12]. Substantial evidences have reflected that metabolic reprogramming contributes to survival of the cancer cells [13], but the relation with metastasis and recurrence in OvCa needs attention. Keeping in pace with the detection of tumor markers, we have assessed by performing IHC the expression of metabolic marker, silent information regulator 1 (sirtuin 1/SIRT1), since dysfunction in metabolic reprogramming is one of the hallmarks of cancer. SIRT1 being NAD+ dependent acts as metabolic sensor, and its deacetylating property hampers cellular metabolism [14]. Recent evidences have suggested the role of extracellular vesicles, the exosomes in metabolic reprogramming of the cancer cells, which promotes their proliferation, angiogenesis, and immunosuppression in the tumor microenvironment (TME) and eventually leads to metastasis [15]. In these efforts, we have tried to evaluate the status of exosomes which are responsible for intercellular communication via transfer of distinct cargoes carried by them and promote peritoneal dissemination [16]. Metabolic alterations have a huge contribution in promoting cancer cell progression and metastasis. When the cancer cells migrate from their primary tumor sites to a secondary location, they need to adapt with the metabolic state in the new metastatic niche. Metabolic factors including several amino acids like asparagine, proline, and serine and several by-products from metabolic pathways like pyruvate, acetate, and lactate as well as the by-products of fatty acid oxidation (FAO) aid the cancer cells to adapt to the metabolic environment of the new TME by supplying energy [17]. Secondly, the acidification of extracellular space due to the release of CO_2_, lactate, and other organic acids from metabolically active cancer cells also contributes in degrading extracellular matrix, thereby promoting metastasis [18]. Furthermore, the accumulation of glycogen in the cancer cells has introduced a new arena of research. The dysregulations of the enzymes involved in glycogen metabolism have been related to tumor cell proliferation, migration, and invasion, which eventually lead to malignancy of the cancer [19]. Recent study has revealed that OvCa cell metabolism requires more FAO in comparison to aerobic glycolysis for increasing the availability of ATP to nourish the cancer cells for proliferation and migration [20]. Glycogen fuels glycolysis to nurture the cancer cells during nutrient deficiency and metabolically compromised conditions in the TME [21]. An oxygen tension level always prevails in the tumor condition leading to a hypoxic atmosphere that can alter cancer cell metabolism as well as induce elevated secretion of the exosomes [22, 23]. As a mode of survival in hypoxic TME, the cancer cells accommodate themselves in small vasculatures of size 100-200 *μ*m which in turn regulates the oxygen limit in the tissues [24]. Cells can directly sense oxygen level that influences cellular physiology by the aid of several regulatory elements [25]. One such controlling element is hypoxia inducible factor 1*α* (HIF-1*α*) which induces the hypoxic TME and is associated with tumor cell metabolism and exosome release. Thus, HIF-1*α* is also taken under consideration in order to develop a compact coordination among the factors considered in this study.

Therefore, in this cohort study, we have analyzed the serum levels of CA-125 at the diagnostic stage and related it to other clinical parameters, and depending on the significant correlated clinicopathological parameters, we have also analyzed the expression of metabolic and metastatic biomolecules which can be further established as therapeutic targets and biomarkers for early diagnosis.

## 2. Materials and Methods

### 2.1. Antibodies and Reagents

Primary antibodies were applied to analyze the expression of SIRT1, HIF-1*α*, CD81, and CD63. Rabbit polyclonal anti-human SIRT1 antibody (Cell Signaling Technology: D739) was used to detect the expression in both normal and tumor samples. For evaluating the expression of HIF-1*α*, we have used rabbit anti-human polyclonal antibody (Santa Cruz Biotechnology: sc10790) and CD81 expression was evaluated using purified mouse anti-human CD81 antibody (BioLegend: #349501). Flow cytometric analysis was observed by anti-human CD63 antibody (BioLegend: #353027). Mouse anti-rabbit IgG-HRP secondary antibody (Santa Cruz: sc-2357) was utilized against the primary antibody. Other reagents used are included in Supplementary file 1.

### 2.2. Study Population and Data Collection

The study population includes women who attended the Cancer Detection Centre at Chittaranjan National Cancer Institute, Regional Centre, Kolkata, India. The study has been approved by the Institute Ethics Committee (CNCI-IEC-40104). The data set comprises clinical characteristics including age, BMI, potent tumor markers like carcinoembryonic antigen levels (CEA), CA-125 and cancer antigen 19.9 (CA 19.9), ascites level, malignancy present in ascites, degree of spread of the disease, differentiation of tumor cells, tumor type, FIGO stage of tumor, TNM grading, laterality of tumor, and size of tumor. The relevant clinicopathological characteristics have been collected from their clinical records. All the information was recorded by the attending physicians.

### 2.3. Study Design

This retrospective study comprises data collected from the records of the patients in the year 2019-2021. Our study includes all the patients (*N* = 248) who have been diagnosed with OvCa irrespective of the age criteria. Patients who have undergone or not undergone surgery and/or chemotherapy with variable clinical characteristics have been considered in the study. The exclusion criteria include those patients who had comorbidities like cardiac problem or gastrointestinal problems, those who were pregnant or lactating, and those who had prior diagnosis of different forms of cancer. Along with the analysis of clinicopathological parameters retrospectively, we were also inclined to evaluate the expression of biomolecules related to metabolic and metastatic regulations in OvCa irrespective of the histological subtypes in order to get the molecular status in patients (*N* = 21).

### 2.4. Patient Sample Collection

OvCa samples were collected from patients who have undergone surgery at Chittaranjan National Cancer Hospital. Tumor tissues and adjacent normal tissues were collected from patients during surgery according to the segregation of CA-125 level at the diagnostic stage with written consent form from the respective patients. The clinical records were collected retrospectively, thus, the written consent information was not required from the patients and prior to analysis, patient details were anonymised.

### 2.5. Immunohistochemical Analysis

Paraffin embedding and tissue sectioning from both tumor tissues and adjacent normal tissues were conducted and IHC staining was followed. The tissue section slides were deparaffinized using xylene followed by rehydration in a series of graded concentrations of alcohol. In order to augment the expression of antigen, 10 Mm citrate buffer solution (pH 6.0) was added to the slides. The slides were further treated with 3% hydrogen peroxide dilute in 100% methanol and kept at room temperature for 15 min, followed by a PBS wash to stop the endogenous peroxidase activity. The slides were kept at 4°C overnight after addition of primary antibody in humidified chambers. A 1 : 500 dilution of SIRT1, HIF-1*α*, ki-67, and CD81 was used as the primary antibodies. After overnight incubation, the slides were washed in PBS and anti-rabbit secondary antibody in a dilution 1 : 1000 was added, followed by incubation at room temperature for 2 hours. The reaction was visualised by adding 3,3′-diaminobenzidine tetrahydrochloride, which reacted for 10 minutes. Finally, haematoxylin was added to counterstain the specimen and dehydrated using a gradient alcohol wash, followed by sealing the slides with coverslips using DPX [26]. The images for each sample were taken in a brightfield compound microscope (Leica Microsystems: #Model DM1000) and analyzed.

### 2.6. Flow Cytometric Analysis

Firstly, single cell suspension was prepared by collagenase IV treatment to the tissues followed by straining the tissue through a 0.4 *μ*m strainer and then centrifuging at 2000 rpm for 10 min. The supernatants for each sample were discarded and incubated in RBC lysis buffer for 5 min at RT, followed by centrifugation at 400*g* for 5 min and the cells were stored at -80°C in 10% DMEM freezing media for subsequent flow cytometry [27]. The single cell suspension was thawed and centrifuged at 2000 rpm for 5 min. After discarding the supernatant, APC/Fire-tagged CD63 antibody in a dilution of 1 : 500 was added and incubated for 1 h [28]. The stained cells were acquired using a BD LSRFortessa Flow Cytometry (San Jose, CA, USA) and analyzed using FCS Express 7.

### 2.7. PAS Staining for Detecting Glycogen Accumulation

PAS staining was conducted following Chatterjee et al. [29]. Briefly, paraffinized slides were deparaffinized using xylene, and then, the slides were taken in Columbia staining dish for treatment with Carnoy's fixative for 10 min. The slides were rinsed 3 times with distilled water, followed by addition of periodic acid solution for staining (10 min). The slides were again washed 3 times with distilled water. After removal of excessive periodic acid stain, Schiff reagent was added to the slides and kept for 5 min. In order to remove the excess Schiff reagent, the slides were again washed with distilled water followed by their dehydration in ascending alcohol solutions (50%, 70%, 90% and 100%). After the dehydration process, the slides were mounted with DPX and covered with coverslips. Images were taken after 1 h of mounting in Leica brightfield microscope in 20x and 40x magnifications and analyzed using Fiji-ImageJ software.

### 2.8. Statistical Analysis

The statistical analysis of the clinical records was performed using IBM SPSS25 Statistics software. The frequency table and cross tabulation were prepared using this software whereas the graphs for the frequency distribution were prepared using GraphPad Prism software by one-way analysis of variance (ANOVA) and unpaired *t*-test wherever applicable. The chi-square test was performed using GraphPad Prism software (version 5). Image analysis was carried out using Fiji-ImageJ software (https://imagej.net/Fiji). Statistical analysis of the clinical images was conducted using GraphPad Prism software. Flow cytometry analysis was performed using FCS Express 7 software. Cox-regression analysis and ROC analysis were done using IBM SPSS25 Statistics software. *P* value < 0.001 and <0.05 was considered statistically significant.

## 3. Results

### 3.1. Analysis of Clinicopathological Data of OvCa Patients' Cohort

A total of *N* = 248 case records have been found eligible to be included in the study.  Table 1 comprises frequency distribution of the clinicopathological data of the ovarian cancer patients. The parameters associated with OvCa including age, levels of tumor markers like CA-125, CEA, and CA 19.9, ascites level, ascites malignancy, degree of dissemination, tumor differentiation, tumor type, FIGO stage, TNM staging, laterality of tumor, and tumor size were considered for the study. Ovarian cancer seems to be more prevalent in patients belonging to the age group 41-60 years (54.8%), and the least occurrence was observed in the age group below 18 years (2.4%), whereas age group 19-40 and greater than 60 years shared the same proportion of patients (21.4%). BMI was observed to be associated with occurrence of the disease where overweight and obese patients (BMI ≥ 25 kg/sq.m) were more likely to develop ovarian cancer (51.2%) as compared to the normal (38.7%) lying within 18.5 kg/sq.m-24.9 kg/sq.m and underweighted patients (10.1%) whose BMI was <18.5 kg/sq.m. The levels of antigen markers like CA-125, CEA, and CA 19.9 have been associated with diagnosis of the disease [30]. Most of the patients had CEA levels ≥ 5 ng/ml (56.9%) whereas 43.1% of victims had CEA levels < 5 ng/ml. CA-125 has been subdivided into four groups in order to derive the distribution of patients at different levels of CA-125. ≤35 U/ml is considered to be a normal range, but still this level consisted of 12.1% of patients with OvCa. The maximum patients who were diagnosed with cancer were in the range 35.1-499.9 (39.9%), followed by ≥1000 U/ml and 500-999.9 U/ml with 28.2% and 19.9%, respectively. CA 19.9 level greater than ≥28 U/ml was associated with maximum number of patients (68.5%) and 31.5% of patients had CEA levels < 28 U/ml, which is considered to be normal. All the patients considered for the study were associated with different ascites levels determined upon radiological findings. 39.1% of the patients had high levels of ascites, followed by 33.5% having moderate levels and mild ascites were found in 27.4% of the patients. Malignant ascites has been associated with prognosis of the disease leading to poor quality of life and leads to mortality. In our study, we had deduced that more than 50% of the patients had ascites malignancy and 45.2% of the patients had no malignant ascites. Since OvCa has been associated with tumor metastasis or degree of dissemination, it was observed that pelvic dissemination (PD) was recorded to the highest (41.5%), followed by localized tumor (36.3%) and 22.2% was associated with distant metastasis (DM). Differentiation of tumor cells also plays a pivotal role in spread of the disease. Moderately to poorly differentiated OvCa cells were mostly observed in the later stages and associated with significant risk of poor prognosis [31]. Here, it was observed that 50% of the patients had poorly differentiated tumor cells and the remaining half either had well differentiated (23.8%) or moderately differentiated (26.2%) depending on the stage of the cancer. Since EOC is the most common form of OvCa and that was also reflected in our results (93.5%), the remaining 6.5% was of non-EOC. Early diagnosis of OvCa remains the main challenge for survival; thus, it was observed that most of the patients diagnosed were at stage III (29%), followed by stage II (26.2%), stage I (23.4%), and stage IV (21.4%), respectively. The patients were further categorized based on TNM grading where T3 stage (43.5%) with involvement of both the ovaries with peritoneal metastasis, N1 stage (36.3%) with regional lymph node metastasis, and Mx stage (42.7%) where distant metastasis was not observed was maximum shown in Table 1. In respect to laterality of tumor, unilateral tumor was higher (62.9%) than bilateral tumor (37.1%) and tumor size less than <5∗5∗5 ccm (63.7%) was more in comparison to tumor more than ≥5∗5∗5 ccm (36.3%). Graphical representation of frequency distribution with *P* < 0.05 being statistically significant has been presented in Supplementary file 2.

### 3.2. Association of CA-125 Level with Clinicopathological Features

The levels of CA-125 at the time of diagnosis were recorded and categorized into low and high. Low level included CA-125 level ≤ 35 U/ml, and high level (>35 U/ml) was subdivided into three sections as 35.1-499.9, 500-999.9, and ≥1000 U/ml.  Table 2 summarizes the data of patient cohort distributed among the various levels of CA-125. A nonsignificant relation was deduced in case of CA-125 with age (*P* = 0.1968) and other tumor marker levels CEA (*P* = 0.0610) and CA 19.9 (*P* = 0.0836) whereas a strong association of CA-125 was observed with the other clinicopathological parameters like BMI (*P* = 0.0153), ascites level and ascites malignancy, and tumor differentiation (*P* < 0.0001).

### 3.3. SIRT1 Expression Increases in Ovarian Tumor in Association with Overexpression of HIF-1*α* in the Tumor Tissues

**Figure 1:**
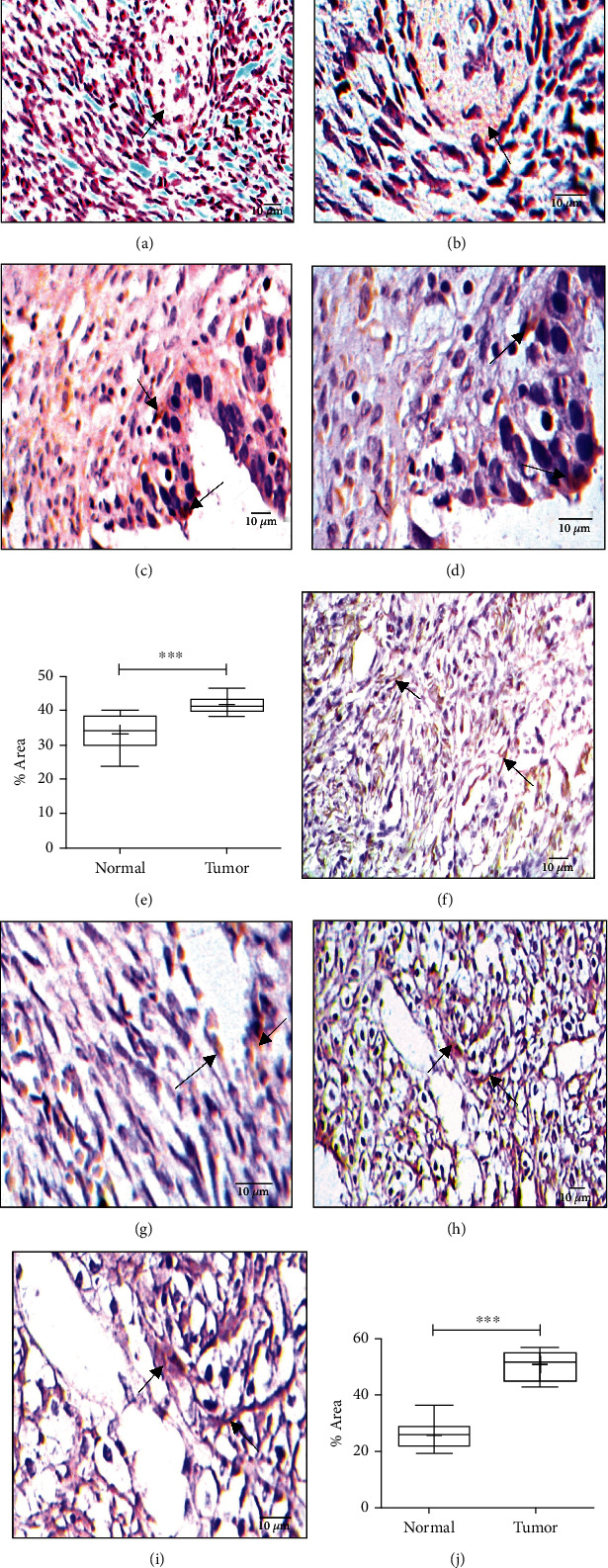
Expression of SIRT1 and HIF-1*α*. (a) SIRT1 expression in normal ovarian tissue (20x). (b) SIRT1 expression in normal ovarian tissue (40x). (c) SIRT1 expression in ovarian tumor tissue (20x). (d) SIRT1 expression in ovarian tumor tissue (40x). (e) Graphical representation of SIRT1 expression, *P* = 0.0003. Expression of HIF-1*α*. (f) HIF-1*α* expression in normal ovarian tissue (20x). (g) HIF-1*α* expression in normal ovarian tissue (40x). (h) HIF-1*α* expression in ovarian tumor tissue (20x). (i) HIF-1*α* expression in ovarian tumor tissue (40x). (j) Graphical representation of HIF-1*α* expression, *P* < 0.0001.

We have evaluated the metabolic biomarker SIRT1 expression in the ovarian tissue both in cancerous state and normal state. It was observed that SIRT1 expression was remarkedly higher in the cancer tissue in comparison to the adjacent normal ovarian tissue (*P* = 0.0003). SIRT1 is the downstream target of HIF-1*α* which is one of the major factors to create a hypoxic TME, contributing to tumor development and progression. In order to determine the nonphysiological oxygen tension level (hypoxia) in the TME, we have evaluated the level of expression of HIF-1*α* in both cancer and normal tissues. HIF-1*α* expression was significantly high in cancer tissues in comparison to normal tissues (*P* < 0.0001). Moreover, a decreased expression of HIF-1*α* with reduced SIRT1 expression in the normal tissues further validated the fact that hypoxic TME created by HIF-1*α* regulates the expression of SIRT1 in the cancer tissues with a significant correlation (Figures 1(a)–1(j)).

### 3.4. Overexpressed SIRT1 in TME Induces Greater Secretion of Exosomes from OvCa Cells

**Figure 2:**
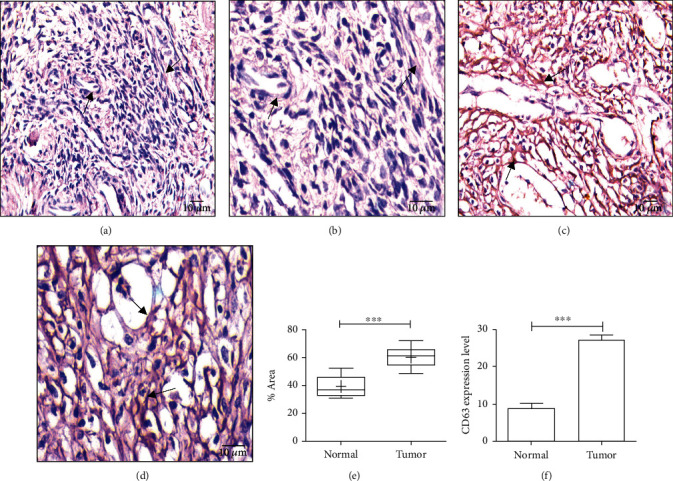
Expression of CD81 and CD63. (a) CD81 expression in normal ovarian tissue (20x). (b) CD81 expression in normal ovarian tissue (40x). (c) CD81 expression in ovarian tumor tissue (20x). (d) CD81 expression in ovarian tumor tissue (40x). (e) Graphical representation of CD81 expression, *P* < 0.0001. (f) Flow cytometric graphical representation of CD63 in normal and tumor tissues (*P* < 0.0001).

We have performed IHC analysis for exosomal surface marker CD81 in OvCa tissues as exosomes participate in preparing a premetastatic niche (PMN) for the migrating cancer cells at the secondary site and promote metastasis [32]. We observed an elevated expression of CD81 in the cancer tissues as compared to normal tissues (Figures 2(a)–2(d)), and the significant graphical representation is found in Figure 2(e) (*P* < 0.0001). Further, to validate the IHC result, flow cytometric analysis for exosomes detection with CD63 antibody depicted increased levels of CD63 in the cancer cells in comparison to the normal cells (Figure 2(f)).

### 3.5. Overexpression of SIRT1 and Exosomes Influences the Metastasizing Capacity of the OvCa Cells

**Figure 3:**
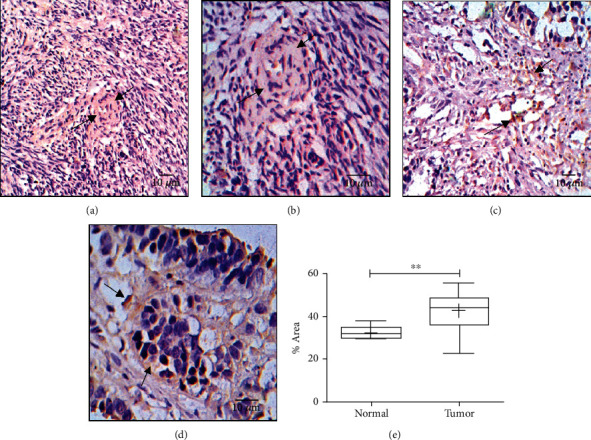
Expression of ki-67. (a) ki-67 expression in normal ovarian tissue (20x). (b) ki-67 expression in normal ovarian tissue (40x). (c) ki-67 expression in ovarian tumor tissue (20x). (d) ki-67 expression in ovarian tumor tissue (40x). (e) Graphical representation of ki-67 expression, *P* = 0.0034.

Imbalance in the SIRT1 expression and exosome concentration shows a positive correlation with the proliferation marker, ki-67. IHC was conducted to evaluate the metastasizing ability of the OvCa cancer cells (Figures 3(a)–3(e)); it was observed that expression of ki-67 was significantly higher in the tumor cells as compared to the normal cells (*P* = 0.0034). This indicated that the chances of metastasis were enhanced when SIRT1 is overexpressed which in turn influences the exosomes concentration in the tumor cells.

### 3.6. Dysregulated SIRT1 Influences the Deposition of Glycogen in OvCa

**Figure 4:**
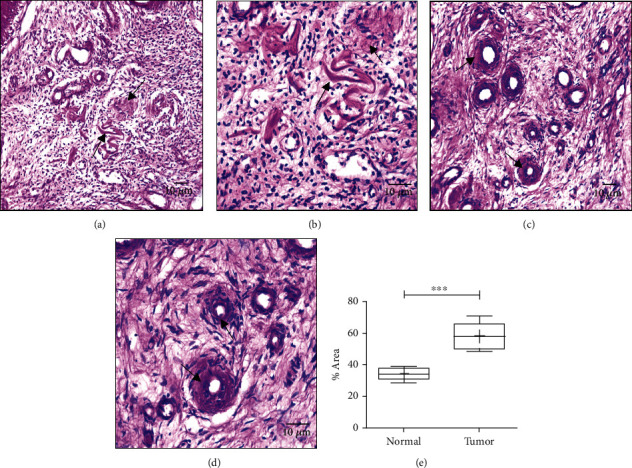
Levels of glycogen deposition. (a) Glycogen deposition in normal ovarian tissue (20x). (b) Glycogen deposition in normal ovarian tissue (40x). (c) Glycogen deposition in ovarian tumor tissue (20x). (d) Glycogen deposition in ovarian tumor tissue (40x). (e) Graphical representation of glycogen deposition, *P* < 0.0001.

Deposition of glycogen serves as nutritional source for the survival of cancer cells; thus, periodic acid/Schiff (PAS) staining was conducted to determine the accumulation of glycogen in both the tissues which revealed that the glycogen deposition was remarkably more in the cancer cells than in the normal cells (*P* < 0.0001), illustrating that the cancer cells have huge storage of glycogen for their usage during nutrient deprivation stage for their growth and survival (Figures 4(a)–4(e)).

### 3.7. Correlation between Clinical Factors and Overall Survival

**Figure 5:**
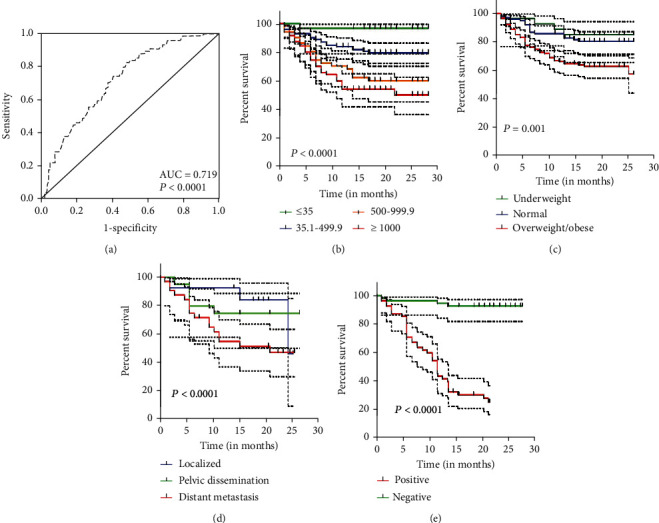
ROC curve and Kaplan-Meier overall survival (OS) graphs of clinicopathological parameters. (a) ROC curve of CA-125. (b) OS graph of CA-125. (c) OS graph of BMI. (d) OS graph of degree of dissemination. (e) OS graph of ascites malignancy.

Survival analysis was conducted with clinically defined endpoint, i.e., OS. Kaplan-Meier plots upon log rank test revealed OS of CA-125 (*χ*^2^ = 26.841) was significant (*P* < 0.0001) represented in Figure 5(b). Similarly, OS of both degree of dissemination (*χ*^2^ = 85.033) and ascites malignancy (*χ*^2^ = 45.042) and BMI (*χ*^2^ = 13.473) had shown significance at *P* < 0.0001 and *P* = 0.001, respectively (Figures 5(c)–5(e)). The overall mean survival time for all the factors was observed to be 18.780 months with 95% CI 17.754–19.805. The ROC curve of CA-125 (Figure 5(a)) has been evaluated which revealed an area under curve (AUC) of 0.719 which was statistically significant (*P* < 0.0001). The best cutoff point for CA-125 in this study is 327.7 U/ml with sensitivity = 82.4% and specificity = 52.3%.

### 3.8. Risk Attributes of Clinicopathological Parameters in Survival of OvCa Patients

Depending on the correlation of CA-125 with the clinicopathological characteristics, we have performed overall survival analysis of the patients in relation to BMI, ascites malignancy, degree of dissemination, tumor differentiation, FIGO stage and tumor size. Univariate analysis revealed BMI, CA-125, ascites level, ascites malignancy, tumor differentiation, degree of dissemination, FIGO stage, tumor type, T stage, N stage, M stage, laterality, and tumor size (Supplementary file 3). Based on these findings, multivariate Cox-regression analysis was conducted.  Table 3 unveils the multivariate Cox regression analysis of the parameters taken under consideration in which BMI (HR 1.759; 95% CI 1.156–2.677; *P* = 0.008), ascites malignancy (HR 0.336; 95% CI 0.124–0.911; *P* = 0.032), and degree of dissemination (HR 1.994; 95% CI 1.251–3.178; *P* = 0.004) are independent predictors of the OS of the patients.

## 4. Discussion

Early diagnosis of OvCa, which helps to improve the survival rate of the patients, remains a challenge for the clinicians. However, CA-125 level serves as a promising diagnostic marker, but still the survival of the patients remains compromised in most of the cases. Thus, there is a need to develop personalised treatment strategy for the patients for better response. This research work has analyzed the CA-125 levels and correlated with the clinicopathological parameters. Since most of the parameters held a significant correlation, we have targeted only those parameters that were related to metabolism like BMI and the spreading of cancer like degree of dissemination (metastasis) that hold the risk for the survival of the patients. Thus, we were more inclined to analyze the expression level of SIRT1, ki-67, status of glycogen deposition, HIF-1*α*, and intercellular communicators, i.e., exosomes, so that a preliminary idea can be provided for designing the individualized treatment regimen by targeting them at the diagnostic level. Upon further investigation and validation, they can be established as biomarkers for OvCa.

Metabolic reprogramming is essential for the cancer cells to facilitate cellular growth, proliferation, and their persistence. Current researches on energy metabolism in OvCa have also been steered, where 39 energy metabolism-related genes significantly correlated with disease prognosis [33]. SIRT1, a class III histone deacetylases (HDACs), is considered as the key metabolic regulator. In response to various external stimuli, it impacts the metabolic status of the cancer cells in regulating the chromatin structure and gene expression [34]. Among the seven members of the sirtuin family, SIRT1 is the most extensively studied in the cancer types as there are conflicting evidences regarding the association of SIRT1 and tumorigenesis [35]. For instance, in colon cancer and melanoma, it acts as a tumor suppressor by deacetylating histidine triad nucleotide-binding protein (HINT) 1 and enhances its binding efficacy with oncogenic transcription factor *β*-catenin and microphthalmia transcription factor (MITF) [36]. Simultaneously, in lung adenocarcinoma, it induces the tumorigenicity of the cancer cells, thus fuelling the cancer progression and survival of the cancer cells [37]. Activity of SIRT1 is tightly regulated by NAD+/NADH levels in a cell, where for deacetylation of targeted proteins, NAD+ serves as a major substrate for SIRT1 [34]. Evidences suggest that hypoxia is one of the hallmarks of malignant tumors and SIRT1 being a downstream target of HIF-1*α* is observed to be upregulated in OvCa, thereby conferring cancer stem cell-like properties [38]. HIF-1*α* expression can be a prognostic biological marker in poorly differentiated serous ovarian carcinoma [39]. Our data revealed that HIF-1*α* was expressed more in the OvCa tissues as compared to the adjacent normal ones, which might have regulated the expression of SIRT1 which could influence glycogen deposition in the cells [40]. Interestingly, hypoxic TME also facilitates greater secretion of minute extracellular biomolecules especially exosomes which aids in the promotion of the aggressiveness of cancer cells [41]. In an earlier study, it has been established that CA-125 production and release are related to exponential cell growth. Inhibition of the cell cycle at the G2/M phase by cycloheximide not only resulted in the death of the cells but also significantly minimized the rate of secretion of CA-125 from the cancer cells [42]. The secretion of CA-125, a heavily glycosylated membrane-bound protein, relies upon the epidermal growth factor receptor (EGFR) signal transduction pathway signaling of the cancer cells [43], and prior to its release, it undergoes phosphorylation at the serine/threonine followed by its cleavage by extracellular protease [44]. In addition to this, Li et al. have also established a link between SIRT1, EGFR, and BRCA1 axis in enhancing the resistance of the OvCa cells towards cisplatin. They have observed that the expression levels of SIRT1, EGFR, and BRCA1 were significantly high in the cisplatin-resistant OvCa cells as compared to the cisplatin-sensitive cancer cells [45]. Since an underlying signaling cascade involving BRCA1-mediated SIRT1 transcriptional regulation of EGFR expression has been evaluated, it validates our hypothesis that increased SIRT1 expression directly modulates the CA-125 levels. Thus, estimating the SIRT1 levels at the diagnostic level can also be established as a metabolic biomarker of OvCa. In addition, the association of SIRT1 and exosomes has been also deduced by Latfikar et al. illustrating that SIRT1 exhibits a tumor suppressive role in breast cancer and reduction in SIRT1 expression promotes elevated secretion of exosomes [46]. It is definite that SIRT1 influences the release of exosomes, but according to our data, it may be said that SIRT1 has tumor promotive function by inducing greater secretion of exosomes in OvCa [47], which can be estimated by observing its relatively higher expression in the tumor tissues as compared to the normal ones. Recently, it has been evaluated that exosomal CA-125 increases OvCa diagnostic efficacy [48]. This finding further confirms that greater concentration of exosomes can be related to higher CA-125 levels. CA-125 being synthesized by the epithelial cells [49] may get incorporated into the exosomes to be secreted during their cargo loading. Since the hypoxic state of TME enhances the production of exosomes, it can be established that hypoxic condition mediated by HIF-1*α* regulates the expression of SIRT1 which in turn enhances the secretion of CA-125 and exosomes. Exosomes, being 30 nm-150 nm in size, have the capability to cross all the biological barriers and migrate to different parts of the body [50]. So, these exosomes carrying CA-125 may be assessed easily in the serum of the patients. Exosomes perform both protumorigenic and antitumorigenic roles in cancer [51]. Furthermore, the increase in levels of CA-125 by 5 U/ml, within the normal range, after the treatment has been observed to be associated with the recurrence and survival in OvCa patients [52]. This finding paves a new way in analyzing the risk of recurrence by estimating the concentration of exosomes carrying CA-125 present in the patients' blood, at a gap of 3-6 months, following the prescribed treatment, thus establishing it as post-treatment biomarker for OvCa. Moreover, elevated level of exosomes can itself be considered as a potent biomarker and therapeutic target in OvCa patients [53]. Exosomal cargoes have also been reported to influence energy metabolism such as in breast cancer exosomal miR-122 induces metastasis by reprogramming glucose metabolism in PMN thereby providing nutrient for the cancer cells [54]. Apart from being a diagnostic tool, exosomes may also participate in SIRT1-driven energy metabolism in OvCa patients and promote metastasis. The distinguished cross-talks between the exosomes and other cells in the TME potentiate the host cells to acquire metastasizing capability which in turn show a direct negative impact in the survival of such patients [55]. In colorectal cancer, it has been observed that under hypoxia the exosomes derived from hypoxic tumor cells when internalized by the normoxic cells impart a metastatic phenotype to the recipient cells. These exosomes transfers Wnt4 depending on HIF-1*α* which in turn activates the *β*-catenin signaling pathway and facilitates metastasis, contributing to cancer progression [56]. Similarly, an investigation conducted by Abdouh et al. has stated that exosomes derived from the sera of cancer patients transmitted potent cargoes which influence the recipient cells to acquire malignant traits. Upon IHC of serum-derived exosome-treated cells, a positivity of 85-90% ki-67 has been observed in the ovarian cells along with remarkable increase in other malignancy markers like PAX8 and WT1 [57]. This confirmed the fact that exosomes carry ki-67 and transfer it to the recipient cells to enhance metastasis. According to our investigation, increased concentration of exosomes indicates that the metastasizing potential of the cancer cells is enhanced, validated by greater expression of ki-67 in tumor tissues.

It was observed that 5.5% of all cancer types in UK were related to overweight and obesity [58]. In 2007 and 2008, a meta-analysis in 28 populations and 12 cohort studies, respectively, demonstrated that the obese patients were at a greater risk of developing OvCa [59]. In India, there is a higher prevalence of obesity which affects more than 135 million people [60] because of rapid urbanization. From this study, it has been deduced that a greater proportion of patients are obese and overweight which has interestingly corroborated with the fact that obesity and OvCa are associated with each other. The modulations in metabolism of obese individuals increase the risks of developing the disease. Moreover, evidences have revealed that obesity induces metabolic changes which can also contribute to the increased levels of CA-125 [61]. Since obesity is one of the risk factors in OvCa development, understanding the baseline metabolic alterations leading to obesity is essential to develop targeted potential therapy. Evidences have suggested that the demand for glucose in TME is higher for the survival of the cancer cells. So, we were inclined to analyze the level of glycogen deposition in the cancer cells which serves as a storage for glucose to be utilized for increased glycolysis. However, it has been well established that obesity redirects glucose metabolic flux which in turn induces glycogen deposition in human adipocytes [62]. Glycogen deposition is not only limited to adipocytes, rather hypoxia induced by HIF-1*α* has been reported to promote glycogen deposition in human ovarian clear cell carcinoma cells [63]. Our data has revealed the distinct level of glycogen deposition between the adjacent normal and cancerous ovarian tissue, inferring that the serum CA-125 levels can also provide a lead to metabolic regulations of OvCa glycogen deposition. Although in depth, further investigations are necessary for the biomarkers related to metabolic alterations in OvCa.

Statistical analysis from data of the patients' cohort revealed that different levels of CA-125 in OvCa patients are significantly correlated with all the clinicopathological parameters except the age, CEA, and CA 19.9 levels. Thus, CA-125 can be a prognostic factor for patients with OvCa. From the IHC data, we can hypothesize that, under hypoxic TME created by upregulation of HIF-1*α*, the expression level of SIRT1 is elevated which may be correlated with the increased levels of exosomes in the TME and also promotes greater accumulation of glycogen in the tumor tissues as compared to the normal tissues. The key findings from this preliminary data of our research are based on the clinicopathological correlation of serum marker CA-125 with the clinicopathological factors which has provided a lead in the study to analyze the biomolecules in order to establish new targets for OvCa treatment.

## 5. Conclusion

This comprehensive study has correlated serum CA-125 levels with the clinicopathological parameters indicating that CA-125 apart from a diagnostic element can also be a prognostic factor that can provide a perception on the metastatic and probability of recurrence in the OvCa patients. This reflected a number of significant outcomes both in analyzing the clinicopathological parameters and in the clinical analysis. In addition to it, CA-125 level variations can also be implemented to detect the dysregulations in the cancer cell metabolism and concentrations of exosomes. The disruption in the normal balance of metabolic pathways as well as elevated metastatic regulations can be responsible for significant level of CA-125. Since a better diagnostic and prognostic factor is essential in the treatment strategy of OvCa, the evaluation of the expression level of biomolecules like SIRT1, HIF-1*α*, glycogen deposition, and exosome level provides a preliminary idea to target the mechanisms involving them for a better therapeutic outcome for the OvCa patients. According to our study, since CA-125 levels can aid in depicting the metabolic rate and risk of metastasis, individualized treatment strategies can be planned for better survival of the patients by targeting the biomolecules related to metabolism and metastasis. Further intensive explorations are essential in this new arena of research for detecting the underlying invloved biomolecules and signaling cascades, driving the increased metabolism rate and risk of metastasis, which can be further established as potent biomarkers for OvCa.

## Figures and Tables

**Table 1:** Frequency profiles of clinicopathological parameters assessed in the study subjects.

Clinicopathological parameters	Frequency (*N*)	Percentages (%)
*Age*		
≤18	6	2.4
19-40	53	21.4
41-60	136	54.8
>60	53	21.4
*Body mass index (BMI)*		
Underweight	25	10.1
Normal	96	38.7
Overweight/obese	127	51.2
*CEA (ng/ml)*		
<5	107	43.1
≥5	141	56.9
*CA-125 (U/ml)*		
≤35	30	12.1
35.1-499.9	99	39.9
500-999.9	49	19.9
≥1000	70	28.2
*CA 19.9 (U/ml)*		
<28	78	31.5
≥28	170	68.5
*Ascites level*		
High	97	39.1
Moderate	83	33.5
Low	68	27.4
*Ascites malignancy*		
Negative	112	45.2
Positive	136	54.8
*Degree of dissemination*		
Pelvic dissemination	103	41.5
Distant metastasis	55	22.2
Localized	90	36.3
*Tumor differentiation*		
Well differentiated	59	23.8
Moderately differentiated	65	26.2
Poorly differentiated	124	50.0
*Tumor type*		
Epithelial	232	93.5
Nonepithelial	16	6.5
*FIGO stage*		
I	58	23.4
II	65	26.2
III	72	29.0
IV	53	21.4
*T stage*		
T1	58	23.4
T2	82	33.1
T3	108	43.5
*N stage*		
N0	83	33.5
Nx	75	30.2
N1	90	36.3
*M stage*		
M0	81	32.7
Mx	106	42.7
M1	61	24.6
*Laterality*		
Unilateral	156	62.9
Bilateral	92	37.1
*Tumor size (ccm)*		
<5∗5∗5	158	63.7
≥5∗5∗5	90	36.3

**Table 2:** Association of CA-125 levels with clinicopathological parameters.

Clinicopathological parameters	CA-125 (U/ml)				*χ* ^2^	*P* value
	Low	High			
	≤35	35.1-499.9	500-999.9	≥1000		
*Age*						
<18	1	3	2	0	12.30	0.1968
19-40	8	28	9	8		
41-60	13	50	29	44		
>60	8	18	9	18		
*Body mass index (BMI)*						
Underweight	2	11	3	9	15.73	0.0153
Normal	14	49	17	16		
Overweight/obese	14	39	29	45		
*CEA (ng/ml)*						
<5	14	52	17	24	7.369	0.0610
≥5	16	47	32	46		
*CA 19.9 (U/ml)*						
<28	6	40	13	19	6.658	0.0836
≥28	24	59	36	51		
*Ascites level*						
High	0	15	25	57	135.7	<0.0001
Moderate	5	46	19	13		
Low	25	38	5	0		
*Ascites malignancy*						
Positive	1	39	33	63	79.71	<0.0001
Negative	29	60	16	7		
*Degree of dissemination*						
Pelvic dissemination	3	40	29	31	108.3	<0.0001
Distant metastasis	0	7	13	35		
Localized	27	52	7	4		
*Tumor differentiation*						
Well differentiated	21	32	4	2	94.56	<0.0001
Moderately differentiated	9	34	13	9		
Poorly differentiated	0	33	32	59		
*Tumor type*						
Epithelial	24	92	46	70	14.02	0.0029
Nonepithelial	6	7	3	0		
*FIGO stage*						
I	21	32	3	2	114.2	<0.0001
II	9	34	13	9		
III	0	26	22	24		
IV	0	7	11	35		
*T stage*						
T1	22	31	3	2	87.55	<0.0001
T2	8	39	14	21		
T3	0	29	32	47		
*N stage*						
N0	15	38	16	14	34.97	<0.0001
Nx	14	35	10	16		
N1	1	26	23	40		
*M stage*						
M0	13	31	19	18	53.25	<0.0001
Mx	17	58	14	17		
M1	0	10	16	35		
*Laterality*						
Unilateral	28	69	24	35	22.93	<0.0001
Bilateral	2	30	25	35		
*Tumor size (ccm)*						
<5∗5∗5	3	52	39	64	71.40	<0.0001
≥5∗5∗5	27	47	10	6		

**Table 3:** Multivariate Cox regression analysis of clinicopathological parameters on OS.

Clinicopathological parameters	Overall survival			
	*P* value	HR	95% CI	
			Lower	Upper
Body mass index (BMI)	0.008	1.759	1.156	2.677
Ascites level	0.158	0.616	0.315	1.206
Ascites malignancy	0.032	0.336	0.124	0.911
Degree of dissemination	0.004	1.994	1.251	3.178
Tumor differentiation	0.278	2.052	0.559	7.523
Tumor type	0.960	0.000	0.000	4.006*E* + 185
FIGO stage	0.842	1.074	0.531	2.172
T stage	0.223	0.664	0.343	1.284
N stage	0.068	1.378	0.977	1.945
M stage	0.837	0.959	0.642	1.433
Laterality	0.650	1.123	0.681	1.850
Tumor size (ccm)	0.392	0.594	0.180	1.959

## Data Availability

The data used to support the findings of this study are available from the corresponding author upon request.

## Abbreviations

AUC: Area under curve
BMI: Body mass index
CA 19.9: Cancer antigen 19.9
CA-125: Cancer/carbohydrate antigen-125
CEA: Carcinoembryonic antigen
DM: Distant metastasis
EGFR: Epidermal growth factor receptor
EOC: Epithelial ovarian cancer
FAO: Fatty acid oxidation
HDACs: Histone deacetylases
HIF-1*α*: Hypoxia inducible factor-1*α*
HINT1: Histidine triad nucleotide-binding protein 1
HR: Hazard ratio
IHC: Immunohistochemistry
MITF: Microphthalmia transcription factor
NAD: Nicotinamide adenine diphosphate
OS: Overall survival
OvCa: Ovarian cancer
PAS: Periodic acid/Schiff
PD: Pelvic dissemination
PMN: Premetastatic niche
SIRT1: Silent information regulator 1
TME: Tumor microenvironment.

## Supplementary Materials

Supplementary 1 List of reagents.

Supplementary 2 Graphical representation of frequency profile of clinicopathological parameters in the patient cohort (*N* = 248).

Supplementary 3 Univariate Cox regression analysis of clinicopathological parameters.
