# Supplementary material for: Understanding the Correlation between Metabolic Regulator SIRT1 and Exosomes with CA-125 in Ovarian Cancer: A Clinicopathological Study
Source: Biomed Res Int. 2022 Apr 20;2022:5346091. doi: 10.1155/2022/5346091 (PMC9053760; doi:10.1155/2022/5346091)
Supplement: Supplementary 1 — List of reagents. [file 5346091.f1.docx]

**Additional Table 1.** List of reagents

| **Serial No.** | **Reagent Name** | **Company** | **Catalogue No.** |
| --- | --- | --- | --- |
| 1. | Hematoxylin Solution (Mayer's, Modified) | ABCAM | ab220365 |
| 2. | DAB Substrate | Sigma Aldrich | 11718096001 |
| 3. | Collagenase Type IV, Cls IV | Sigma Aldrich | C4-22 |
| 4. | DPX new | Sigma Aldrich | 1005790500 |
| 5. | o-Xylene solution | Sigma Aldrich | 40201 |
| 6. | Schiff′s reagent | Sigma Aldrich | 1090332500 |
| 7. | Periodic acid | Sigma Aldrich | P0430 |
| 8. | Acetic Acid Glacial extrapure AR, 99.9% | Sisco Research  Laboratories Pvt. Ltd. | 85801 (0129168) |
| 9. | Methanol extrapure AR, 99.8% | Sisco Research  Laboratories Pvt. Ltd. | 65524 |
| 10. | eBioscience™ 1X RBC Lysis Buffer | Invitrogen | 00-4333-57 |
| 11. | Dulbecco′s Modified Eagle′s Medium - high glucose | Sigma Aldrich | D6429 |
