# Supplementary material for: Understanding the Correlation between Metabolic Regulator SIRT1 and Exosomes with CA-125 in Ovarian Cancer: A Clinicopathological Study
Source: Biomed Res Int. 2022 Apr 20;2022:5346091. doi: 10.1155/2022/5346091 (PMC9053760; doi:10.1155/2022/5346091)

**Additional Figure 1.** Graphical representation of frequency profile of clinicopathological parameters in the patient cohort (N=248)


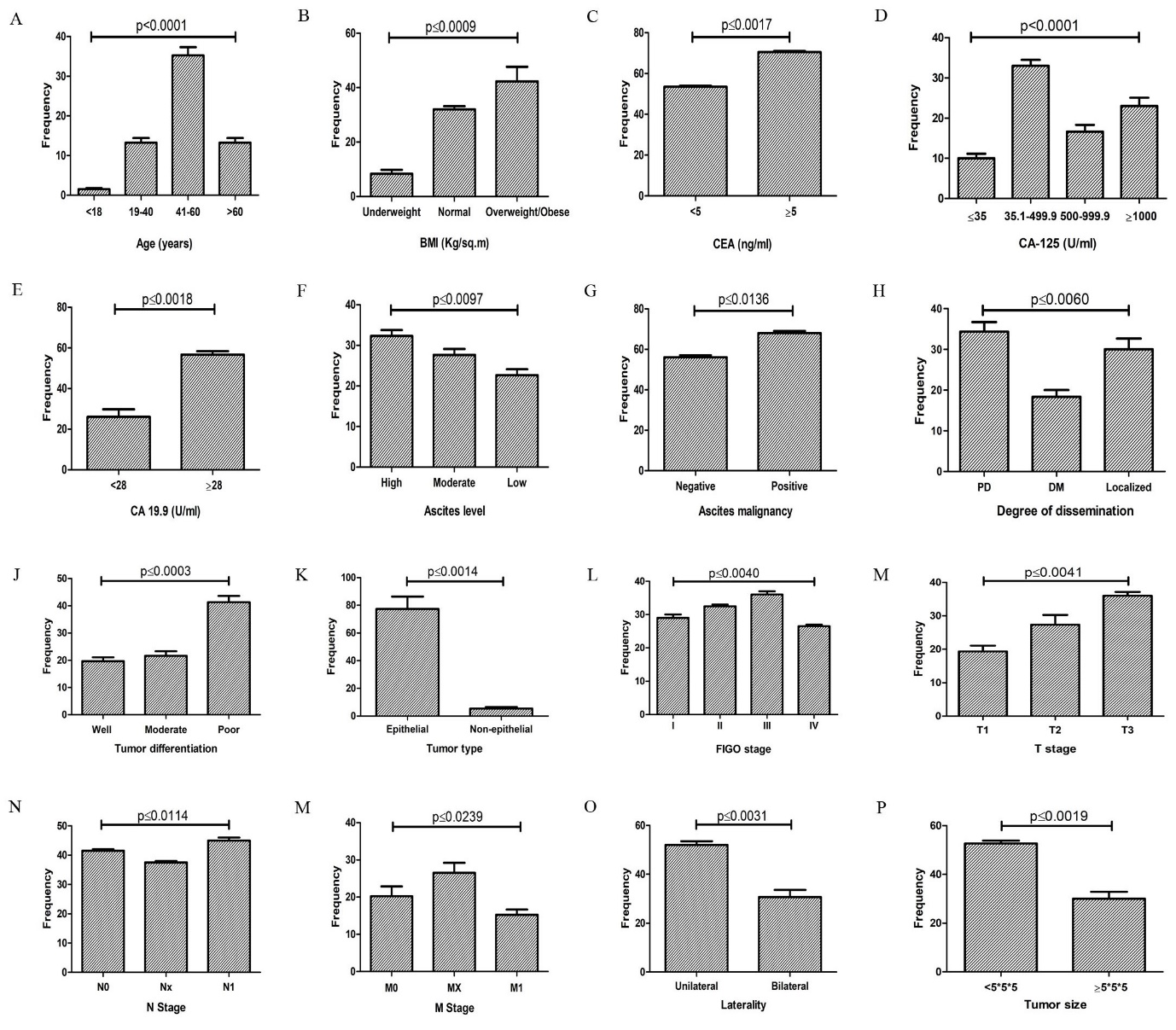

Supplement: Supplementary 2 — Graphical representation of frequency profile of clinicopathological parameters in the patient cohort (N = 248). [file 5346091.f2.docx]
