# Supplementary material for: Understanding the Correlation between Metabolic Regulator SIRT1 and Exosomes with CA-125 in Ovarian Cancer: A Clinicopathological Study
Source: Biomed Res Int. 2022 Apr 20;2022:5346091. doi: 10.1155/2022/5346091 (PMC9053760; doi:10.1155/2022/5346091)
Supplement: Supplementary 3 — Univariate Cox regression analysis of clinicopathological parameters. [file 5346091.f3.docx]

**Additional Table 2.** Univariate cox regression analysis of clinicopathological parameters.

| Clinicopathological parameters | Overall survival | | | |
| --- | --- | --- | --- | --- |
|  | P-value | HR | 95% CI | |
|  | Lower | | | Upper |
| Body mass index (BMI) | 0.001 | 1.957 | 1.302 | 2.940 |
| CA-125 (U/ml) | <0.0001 | 1.839 | 1.441 | 2.346 |
| Ascites level | <0.0001 | 0.304 | 0.207 | 0.448 |
| Ascites malignancy | <0.0001 | 0.146 | 0.075 | 0.285 |
| Degree of dissemination | 0.028 | 0.748 | 0.577 | 0.970 |
| Tumor differentiation | <0.0001 | 3.718 | 2.335 | 5.921 |
| Tumor type | 0.106 | 0.044 | 0.001 | 1.943 |
| FIGO stage | <0.0001 | 2.566 | 1.966 | 3.350 |
| T stage | <0.0001 | 2.589 | 1.783 | 3.760 |
| N stage | <0.0001 | 2.242 | 1.627 | 3.089 |
| M stage | <0.0001 | 2.228 | 1.600 | 3.103 |
| Laterality | <0.0001 | 2.357 | 1.486 | 3.739 |
| Tumor size (ccm) | <0.0001 | 0.149 | 0.068 | 0.324 |
